# General Overview and Diagnostic (Imaging) Techniques for Neurogenic Thoracic Outlet Syndrome

**DOI:** 10.3390/diagnostics13091625

**Published:** 2023-05-04

**Authors:** Stijn B. J. Teijink, Niels Pesser, Jens Goeteyn, Renée J. Barnhoorn, Marc R. H. M. van Sambeek, Bart F. L. van Nuenen, Hugh A. Gelabert, Joep A. W. Teijink

**Affiliations:** 1 Department of Vascular Surgery, Catharina Hospital, 5623 EJ Eindhoven, The Netherlands; 2 Department of Biomedical Technology, University of Technology Eindhoven, 5612 AJ Eindhoven, The Netherlands; 3 Department of Neurology, Catharina Hospital, 5623 EJ Eindhoven, The Netherlands; 4 Division of Vascular and Endovascular Surgery, David Geffen School of Medicine, University of California, Los Angeles, CA 90095, USA; 5 CAPHRI School for Public Health and Primary Care, Faculty of Health, Medicine and Life Sciences, Maastricht University, 6229 HX Maastricht, The Netherlands

**Keywords:** thoracic outlet syndrome, neurogenic thoracic outlet syndrome, brachial plexus compression, thoracic outlet syndrome care pathway, thoracic outlet syndrome additional imaging

## Abstract

Thoracic outlet syndrome is an uncommon and controversial syndrome. Three different diagnoses can be made based on the compressed structure, arterial TOS, venous TOS, and neurogenic TOS, though combinations do exist as well. Diagnosing NTOS is difficult since no specific objective diagnostic modalities exist. This has resulted in a lot of controversy in recent decades. NTOS remains a clinical diagnosis and is mostly diagnosed based on the exclusion of an extensive list of differential diagnoses. To guide the diagnosis and treatment of TOS, a group of experts published the reporting standards for TOS in 2016. However, a consensus was not reached regarding a blueprint for a daily care pathway in this document. Therefore, we constructed a care pathway based on the reporting standards for both the diagnosis and treatment of NTOS patients. This care pathway includes a multidisciplinary approach in which different diagnostic tests and additional imaging techniques are combined to diagnose NTOS or guide patients in their treatment for differential diagnoses. The aim of the present work is to discuss and explain the diagnostic part of this care pathway.

## 1. Introduction

Thoracic outlet syndrome (TOS) is an uncommon syndrome which describes a spectrum of diseases. Three different diagnoses can be made based on the compressed structure: arterial TOS (ATOS), caused by the compression of the subclavian artery at the scalene triangle; venous TOS (VTOS), caused by the compression of the subclavian vein at the costoclavicular junction; and neurogenic TOS (NTOS), caused by the compression of the brachial plexus or irritation of the scalene triangle or pectoralis minor space. Combinations are possible in the combination of A- and NTOS as well as V- and NTOS [1].

TOS may result from anatomical variations, such as a cervical rib, an anomalous first rib, post-traumatic callus formation on the clavicle and/or first rib, hypertrophic musculature, or a variation in abnormal inserted ligaments [2]. In addition, history of trauma and repetitive arm movements are all described as potential causes for the compression of the neurovascular bundle in the thoracic outlet [3].

NTOS is by far the most common type of TOS, compromising 80–90% of all referred patients [4,5,6]. NTOS patients can present with a variety of complaints such as pain, weakness, paresthesia, and occasionally muscle atrophy. These symptoms can generally be differentiated from VTOS, which accounts for 12% of TOS cases [6]. VTOS patients often present with swelling, pain, distended veins in the upper extremity, and occasionally a bluish color of the arm. In most VTOS patients, symptoms start after a (recent) venous thrombosis in the upper extremity. Only a few patients present with ATOS (1–2%). In patients with ATOS, ischemic manifestations such as pain, coldness, and pallor of the arm with peripheral (micro) embolisms can be seen.

NTOS can be divided into two groups. Patients can present with clear signs and symptoms of muscle atrophy, matching a Gilliatt-Sumner hand (GSH). The second more complex group to diagnose are patients who present without (classic) muscular atrophy of the hand. In these patients, diagnosis is based on history, anamnesis, physical examination, and additional diagnostic imaging to exclude a long list of differential diagnoses (Table 1). Therefore, NTOS is often described as a diagnosis of exclusion, which can result in an extensive and complex diagnostic care pathway.

The diagnostic care pathway for both vascular subtypes of TOS differs significantly from NTOS. Additional imaging in patients suspected of ATOS or VTOS is almost always directive and shows compression and/or signs of damage to the subclavian artery or vein. Unlike vascular TOS subtypes, NTOS is difficult to diagnose since an objective diagnostic test with sufficient sensitivity and specificity is lacking. Moreover, the broad spectrum of NTOS complaints overlaps with many differential diagnoses. This has resulted in inconsistent nomenclature, differences in diagnostic criteria, and a lack of high-quality literature and practice guidelines. Some clinicians still discuss the mere existence of NTOS in contemporary practice. A standardized approach for the diagnosis and treatment of NTOS was made in the reporting standards for TOS of the Society of Vascular Surgery [1]. In this manuscript, we will focus on the diagnosis and treatment of NTOS and the implementation of a care pathway based on the most recent literature and as instituted in our high-volume TOS center.

## 2. History of Neurogenic Thoracic Outlet Syndrome

Stopford and Telford were the first to describe a group of NTOS patients in 1920 [7]. Their description caused a lot of confusion, which resulted in NTOS being refer to as many different names over the years such as Naffziger syndrome, scalenus anticus syndrome, costoclavicular syndrome, fractured clavicle syndrome, cervicobrachial compression syndrome, pneumatic hammer syndrome, first rib syndrome, pectoralis minor syndrome, hyperabduction syndrome, and cervical rib syndrome [6]. In 1970, Roger Gilliatt introduced ‘true’ NTOS based on a strict definition, resulting in NTOS being classified as extremely rare [8]. This definition was later broadened to include ‘disputed’ NTOS, which resulted in even more confusion. NTOS is still a controversial clinical syndrome. Patients regularly tell us that, according to physicians they visited before, NTOS does not exist.

For several reasons, this is an understandable statement. A high complication rate in the 20th century after the surgical treatment of a diagnosis without a clear set of diagnostic criteria may have convinced neurologists that surgical treatment resulted in more morbidity than the original complaints [9,10]. Research performed in those decades was of a low quality (single-center case series) and hard to compare due to the inconsistent use of unclear definitions and poorly defined outcome measures [11].

## 3. Neurogenic Thoracic Outlet Syndrome (NTOS)

NTOS is the collective name for complaints caused by symptomatic compression of the brachial plexus. The etiology of the compression of the brachial plexus in NTOS is controversial and not completely understood. Both congenital and acquired factors are described that increase the chance of developing NTOS. For example, several anatomical variations can narrow the thoracic outlet [12]. Common anatomic variations are cervical ribs, anomalous first ribs, post-traumatic callus formation on the clavicle and/or first rib, hypertrophic musculature, or variations in abnormal inserted ligaments. Additionally, injury can lead to (further) narrowing of the thoracic outlet as a result of fibrosis, scalene and/or pectoralis minor muscle hypertrophy, chronic spasm, or other post-fracture and pathological changes [13].

NTOS patients present with a diverse pattern of complaints in the hand, arm, shoulder, and/or neck as a result of brachial plexus compression. The most common complaints are pain, weakness, paresthesia, and occasionally muscle atrophy. These complaints exist or worsen when patients use the affected arm, especially with overhead movements that stretch the brachial plexus. Repetitive arm movement is also known to cause and worsen NTOS complaints. These complaints often coexist with sensory disturbances and/or loss of strength.

## 4. Gilliatt-Sumner Hand

Gilliatt-Sumner hand (GSH) is a rare presentation of NTOS. Patients with GSH present with atrophy of the mm. abductor pollicis brevis, abductor digiti minimi, and interossei combined with hypesthesia of the n. ulnaris and medial cutaneous antebrachial nerve [8]. This results in numbness, tingling, and/or pain in the medial side of the lower arm and hand [8]. A diagnosis can often be made by a thorough physical examination as the atrophy of the hand muscles follows a characteristic pattern and is combined with the distribution of muscle weakness and sensibility disorders. Due to the relatively low incidence, reported as 1:1,000,000 by Gilliatt [14], symptoms are sometimes incorrectly interpreted as cervical radiculopathy, neuropathy (pressure) of the ulnar nerve, or advanced stage or “atypical” carpal tunnel syndrome.

### Diagnosing GSH

**Figure 1:**
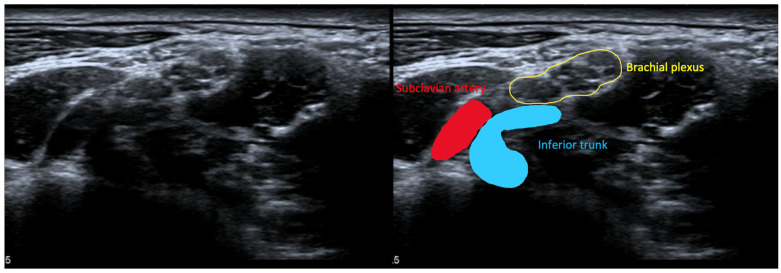
A wedge-sickle sign (blue) seen in high-resolution ultrasound pictures in patients with GSH.

An electromyogram (EMG) can help to diagnose GSH because it often shows brachial plexopathy. A typical EMG shows a lower compound muscle action potential (CMAP) compared to the ulnar nerve and medial nerve and lower sensory nerve action potential (SNAP) of the ulnar nerve and the medial cutaneous antebrachial nerve, in which T1 is more involved than C8 [8]. However, more atypical deviations can be seen in an EMG, such as the increased involvement of C8 compared to T1, or even patterns in which other plexus roots are involved [15]. Some clinicians advocate to first conduct a high-resolution ultrasound (HRUS) of the brachial plexus in NTOS patients because it can visualize the anatomical substrate of the compression and is more patient friendly [16,17]. With this technique, a so-called wedge-sickle sign can be found as evidence for lower brachial plexus compression (Figure 1) [18]. The compression at surgery appears to be a fibromuscular band in most cases.

Treatment of GSH includes thoracic outlet decompression (TOD) to stop the progressive complaints of muscle weakness and pain. A study performed by Goeteyn et al. showed (at least some) clinical improvement in terms of the functional outcome and a decrease in complaints after TOD surgery in a cohort of patients with GSH [17]. Improvement can best be measured using multiple questionnaires (TOS disability scale, CBSQ—cervical brachial score questionnaire, DASH—disability of the arm shoulder and hand, and SF-12—a validated quality of life questionnaire) [17].

## 5. NTOS without Signs of GSH

The vast majority of NTOS patients do not present with signs of GSH. To overcome some of the controversy regarding the diagnosis of NTOS patients without signs of GSH, a group of experts supported by the Society for Vascular Surgery published reporting standards for TOS in 2016. The objective of these standards was to define terminology and standardize definitions to ensure a consensus in terms of diagnosis, severity, and outcome measurements [19,20]. The SVS reporting standards for NTOS state that three out of four of the following criteria should be present in order for the NTOS diagnosis to be made [19]:

1. Local findings include symptoms consistent with inflammation at the scalene triangle. Symptoms of referred pain can be in areas near the inflammation, for example, in the chest wall, axilla, upper back, shoulder, trapezius region, neck, or head. This pain can be triggered by palpation of the affected areas;
2. Peripheral findings include symptoms consistent with nerve compression. This can include numbness, pain, paresthesia’s, vasomotor changes, and weakness. This can be tested using provocative tests that narrow the scalene triangle, an elevated arm stress test (EAST), by stretching the brachial plexus, or by an upper limb tension test (ULTT);
3. The absence of diagnoses of other diseases that could explain these symptoms. For example, cervical radiculopathy, shoulder disease, carpal tunnel syndrome, and complex regional pain syndrome;
4. A positive anterior scalene and/or pectoralis minor test injection.

## 6. Diagnosing NTOS

**Figure 2:**
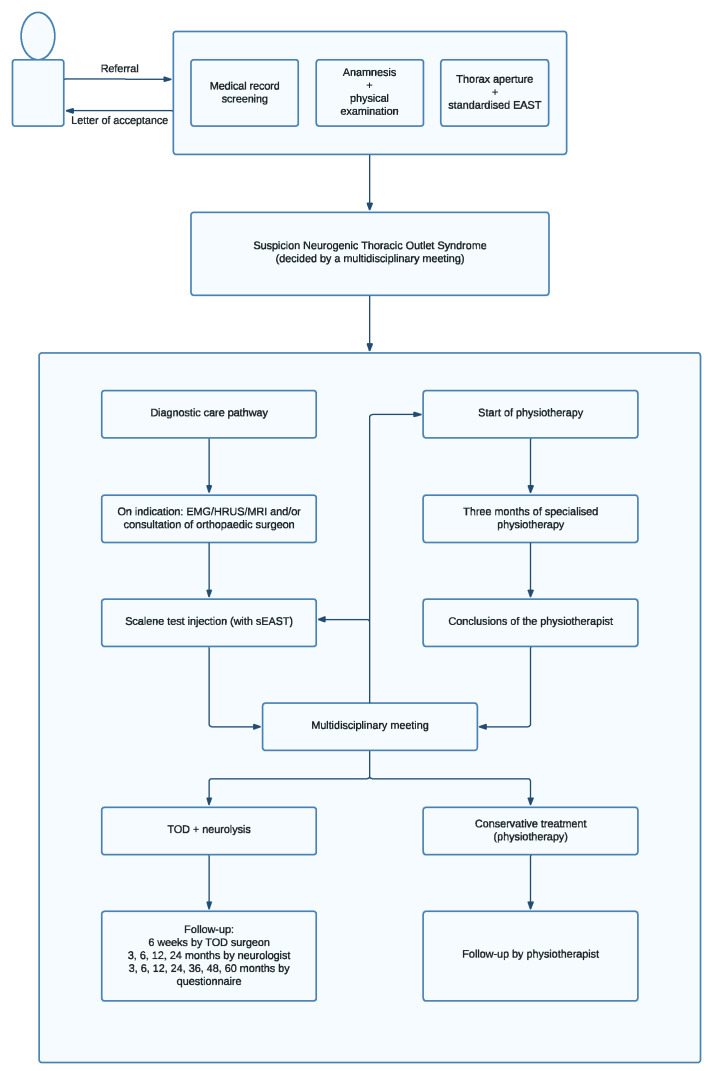
NTOS care pathway.

**Figure 3:**
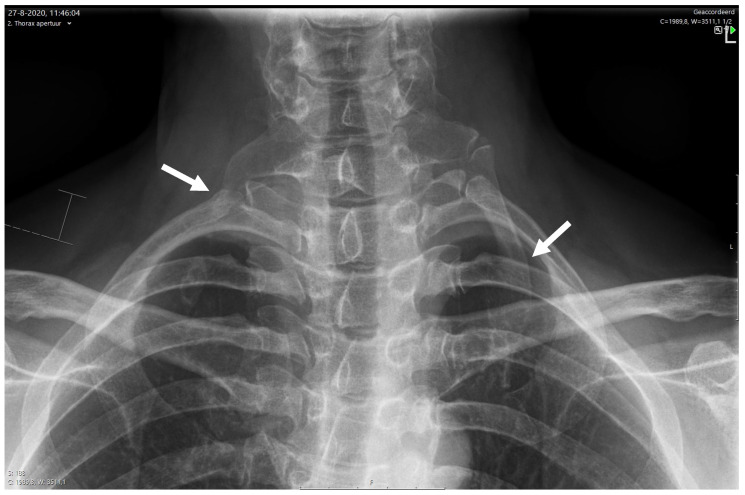
Thorax aperture showing a short RIGHT (white arrow) and a complete left (white arrow) cervical rib with articulation with the first rib on both sides.

The SVS criteria are broad, based on expert opinion, and (ideally) demand the input of several different medical specialties. Therefore, we constructed a multidisciplinary diagnostic and therapeutic care pathway to include all four principles of the SVS reporting standards [21]. Two neurologists, two TOS surgeons, a nurse specialist TOS, several pain anesthetists, an orthopedic surgeon with shoulder expertise, two radiologists, and three physiotherapists with TOS and shoulder expertise work together in this care pathway in daily practice. We make sure that each patient has had (at least) a 3-month period dedicated to TOS physiotherapy before these patients are considered for continuing on our TOS diagnostic care pathway (scalene muscle block) and possible surgery. Our physiotherapists have the autonomous possibility to delay this further investigation and thus stop the TOS care pathway if previous physiotherapy was deemed inadequate or absent. Our NTOS care pathway consists of a diagnostic and therapeutic part (Figure 2). The diagnostic part commences with a thorough history/anamnesis followed by a physical examination. This is conducted separately and independently by one of our neurologists, our TOS nurse specialist, and TOS surgeon. In all cases, additional X-ray diagnostic imaging (Figure 3) as well as a standardized EAST is performed [22,23].

If patients are suspected of NTOS by both the neurologist and TOS nurse specialist/surgeon and 3 months of TOS physiotherapy did not alter their complaints, a scalene muscle block is performed. After completion of this diagnostic care pathway, patients are discussed in a multidisciplinary meeting in which all specialties are present. Together, a treatment plan is made and proposed to the patient in a shared decision-making environment.

### 6.1. Initial Physiotherapy

Conservative (physical therapy) treatment is ideally initiated before visiting our TOS center. NTOS is a complex, inherently unclear, and multifactorial condition that is challenging for physical therapists to treat. Concomitant conditions in the shoulder region are common and make NTOS easy to overlook. The typical NTOS patient experiences centralized neuropathic pain with a musculoskeletal origin. The patient’s history concerning physical therapy and home exercise routines are important to comprehend the tensile and compressive loads being applied to the thoracic outlet [24]. Physical therapy is focused on modifying the patients’ positioning and activities to achieve symptom reduction [25]. This approach can result in the satisfactory alleviation of symptoms in up to 27% of patients [26].

#### 6.1.1. History

The patient’s description of symptoms can be used to determine whether the upper part of the brachial plexus (C5-C7) or the lower part of the brachial plexus (C8-T1) is involved [27]. Lower plexus compression results in symptoms of the ulnar forearm and hand, as well as the axillary and anterior shoulder region. Upper plexus compression results in radiation to the dorsal side of the forearm, wrist, and middle fingers, as well as the chest, periscapular region, and the head. [28]. A large group of patients also experienced autonomic dysfunction of the affected upper extremity. Autonomic dysfunction should not be confused with complaints of arterial thoracic outlet syndrome (ATOS) [29]. However, NTOS can exist in combination with VTOS or ATOS. Different combinations can be distinguished based on history/anamnesis and additional diagnostic imaging modalities such as dynamic venography in four positions for VTOS and computed tomography angiography (CTA) in two positions for ATOS.

According to the diagnostic criteria, NTOS complaints need to exist for at least 12 weeks, which can be seen as a manner to rule out self-limiting complaints. Furthermore, symptoms matching NTOS cannot be related to a single nerve route [30]. Considering the etiology of NTOS, patients are asked about their profession, hobbies, and any sport they take part in, specifically if they use their arms above their shoulders or perform a lot of repetitive movements. In addition, a history of trauma might play a role in causing NTOS. Specifically, mechanisms of injury that may have caused whiplash or had a direct impact on the (upper) arm, first rib, and/or clavicle bone (fractures) are described in relation to NTOS [30,31]. Finally, a medical history consisting of operations in the shoulder area, family-related TOS complaints, daily limitations, and past physiotherapeutic endeavors should be registered [32]. If prior treatment by a physiotherapist was inadequate or not yet performed, the patient is referred to one of our TOS physiotherapists.

#### 6.1.2. Physical Examination

Physical examination starts with localizing the principal sites of the symptoms, identifying factors that cause pain to the hand and arm, and eliciting the degree of neurogenic disability. First, a general neurologic examination is performed, paying special attention to the characteristics of GSH described earlier. A trained physiotherapist investigates and registers the scapulohumeral rhythm, general posture of the upper extremity and shoulders, and possible existence of a scapula alata. A scapula alata or winged scapula is caused by injury/compression of the long thoracic nerve, which can also impact the thoracic outlet region, resulting in NTOS-like complaints [33].

### 6.2. Provocation Tests

Physical examination includes four provocation tests that are advised to be performed in the evaluation of a patient: Morley’s compression test (pressure over MSA/M/scalene triangle), Tinel Sign on that same spot, an elevated arm stress test (EAST), and an upper limb tension test (ULTT). These tests are performed to provoke the complaints that patients experience in day-to-day life. It should be noted that we do not perform the Adson test. A loss of pulse is common in an arm in abduction for a typical NTOS patient since the subclavian artery shares the scalene triangle with the brachial plexus. However, evidence suggests that loss of pulse is also common in ‘healthy’ patients [34,35]. Therefore, it cannot be used as a diagnostic criterion when diagnosing NTOS [29].

#### 6.2.1. Morley’s Compression Test

Morley’s compression test is also called the brachial plexus compression test. Morley’s compression test produces tenderness at the root of the neck in patients with thoracic outlet syndrome [36]. The test is performed with the patient in an upright position with their arms rested at their side. Pressure is placed on the neurovascular structures, the brachial plexus, and subclavian vessels, near the supraclavicular fossa. The test is considered positive when symptoms are reproduced. Symptoms include an aching sensation, tenderness of the root of the neck, and typical localized paresthesia.

#### 6.2.2. Tinel Sign

Tinel Sign provokes recognizable NTOS complaints by palpation and percussion of the medial and anterior scalene muscles and/or pectoralis minor muscle while the patients’ arms rest at their sides. Tinel Sign is tested bilaterally and is positive when there is paresthesia present in the distribution of the median and ulnar nerves [37].

#### 6.2.3. EAST

**Figure 4:**
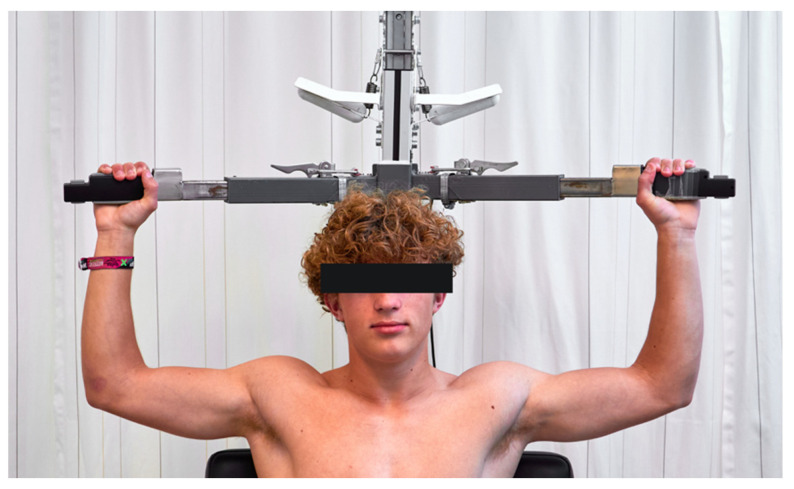
Position during the standardized EAST measurement.

The elevated arm stress test (EAST), also known as the Roos test or positive abduction and external rotation position test, is a provocation test used during physical examination when diagnosing NTOS. The EAST test is a positional test that is thought to ‘provoke’ compression of the brachial plexus. During this test, the patients’ arms are in a 90-degree abduction, external rotation, and their elbows are flexed to 90 degrees while the patient clenches her hands about once every second for three minutes [38]. According to the SVS reporting standards, the test is positive when local or distal pain is present and neurologic symptoms are reproduced [19]. The EAST can also be used to determine the results of a scalene muscle block, in which the outcomes are compared before and after the scalenus muscle block. However, the EAST generates different outcomes, most likely due to deviations in patients’ posture and differences in the instructions provided by physicians. The low test–retest reliability with subsequent varying sensitivity and specificity values reported in the literature endorses the limitations of the EAST [5,22,30,35,39,40]. To overcome the subjective nature of the EAST, we constructed a standardized EAST (sEAST) device in collaboration with the Applied Physics Department of Fontys Academy. This device ensures a correct posture is employed during the test without a practitioner having to compensate, and it automatically generates the test results (Figure 4). In addition to the duration, the sEAST also measures the pinch force of the hand and calculates several power and fatigue parameters. These parameters might be helpful in the diagnostic process considering that others have shown that the pinch force in the symptomatic hand is 19% lower compared to the asymptomatic hand in patients with NTOS. [41] In our validation study, the sEAST shows good test–retest reliability for the duration and grip strength parameters. The good test–retest reliability means that it is good for measuring the effectiveness of a scalenus muscle block, which shall be explained in more detail later [23].

#### 6.2.4. ULTT

**Figure 5:**
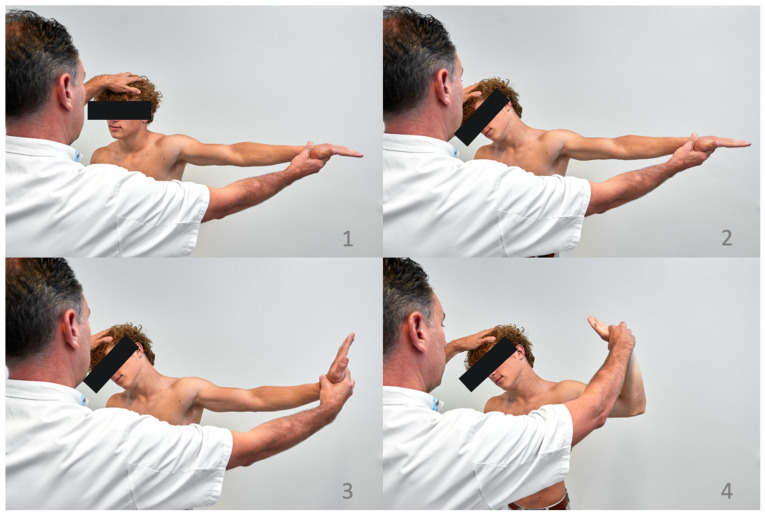
Upper limb tension test. **1**: Straight arm in 90-degree abduction; **2**: the head is tilted to the unaffected/opposite side of the body; **3**: wrist extension; and **4**: elbow flexion to a 90-degree angle.

The upper limb tension test (ULTT) is performed to reproduce symptoms potentially related to brachial plexus irritation by stretching the upper extremity neural axis [1]. The patient is asked to stand and extend their affected arm to 90 degrees abduction with their elbow and wrist straight. This is followed by flexing the neck away from the affected side followed by extending the wrist. The last step is flexion of the elbow to a 90-degree angle (Figure 5) [5]. Symptom reproduction is assessed during the different stages of positioning. The test is positive if symptoms are reproduced and if bending the head to the contralateral side increases symptoms and bending to the ipsilateral side decreases symptoms. A positive ULTT is seen in most of the patients with NTOS [20].

### 6.3. Additional Imaging Techniques

After assessing the patients’ medical history and conducting a physical examination, the degree of suspicion of NTOS can be made, which helps to further determine the diagnostic care pathway. Patient-specific considerations regarding the need for additional imaging using X-ray, magnetic resonance imaging (MRI), EMG, ultrasound, and Duplex ultrasound to exclude differential diagnoses are made in this stage of the diagnostic process. A lot of research has been done to make the NTOS diagnosis based on the outcomes of a single imaging technique. However, a diagnostic imaging technique with a high specificity and sensitivity to diagnose NTOS does not (yet) exist. Therefore, imaging techniques should be used complementary to each other to exclude differential diagnoses and make NTOS diagnosis more likely. The added value of the most used imaging techniques is described below.

#### 6.3.1. X-ray for NTOS

Chest radiography is often used as an initial imaging modality in TOS. To evaluate the bony structures, a separate aperture view is advised. This is an AP (anterior to posterior)-oriented view of the upper thoracic aperture. This specific aperture allows a better evaluation to be performed of bony abnormalities, including cervical ribs, elongated C7 process (which can be suggestive of a scalenus minimus variant), and bony abnormalities of the first rib (congenital anomalies, malunited fractures, Paget disease, tumor, or fibrous dysplasia) [42]. Although rarely found, a chest radiography can also visualize an underlying tumor [42]. An X-ray of the thoracic aperture is made in every patient suspected of NTOS, considering the importance of evaluating bony abnormalities both for the diagnosis and treatment of NTOS.

#### 6.3.2. Magnetic Resonance Imaging for NTOS

Authors suggest that an MRI scan can show asymmetry and edema surrounding the brachial plexus, post-traumatic callus, scarring or deformation, exostosis, accessory fibrous bands, or accessory muscles [43,44]. However, both sensitivity and specificity of the possible abnormalities vary and might not always be explanatory of the NTOS complaints. This might be related to the limitations of posture during an MRI scan as a significant number of patients do not have any complaints when their arms are in a neutral position. One can argue that the compression of the brachial plexus cannot therefore be reliably objectified with an MRI scan as it is important to have the ability to change to provoking positions during imaging. However, MRI is frequently used to exclude a (cervical) radiculopathy in patients suspected of NTOS and is the imaging technique of choice in the evaluation of soft tissue disorders. Further research is necessary to evaluate abnormalities found in MRI scans before it can be considered a reliable, confirmative imaging technique for NTOS.

#### 6.3.3. Electrodiagnostic Study for NTOS

Electrodiagnostic studies can be used to show deviations matching brachial plexopathy. However, NTOS-specific electrodiagnostic findings have proven to be insufficient to diagnose NTOS. Data from our center show that the absence of NTOS-specific findings is of a low diagnostic value with a negative predictive value of 35,9% [15]. This is endorsed by other studies which have found a low rate of NTOS-specific abnormalities in patients suspected of NTOS [45]. However, clinicians should realize that these data are dependent on the used definition of NTOS in these studies, as the groups in which nerve conduction studies are researched within are very heterogeneous. Good results after thoracic outlet decompression surgery in patients without NTOS-specific findings in electrodiagnostic studies also imply that the sensitivity of electrodiagnostic studies in NTOS patients leaves something to be desired. Therefore, an NTOS diagnosis should not be made on the outcomes of electrodiagnostic studies alone. Chances of finding NTOS-specific abnormalities increase if patients present with signs of (GSH) atrophy. Electrodiagnostic studies can be used to exclude common differential diagnoses, such as carpal tunnel syndrome or ulnar neuropathy, and therefore can be used by a neurologist to answer specific diagnostic questions. However, it is not sufficient as a standardized screening method for every patient referred with suspicion of NTOS.

#### 6.3.4. High-Resolution Ultrasound for NTOS

High-resolution ultrasound (HRUS) is an accessible and relatively cheap diagnostic technique, though it is highly operator (experience)-dependent. HRUS should be used in patients with NTOS patients presenting with signs and symptoms of GSH. In these patients, HRUS can be used to identify fibromuscular bands that compress the brachial plexus, the so-called wedge-sickle sign (WSS). The presence of a wedge-sickle sign has a sensitivity of 95% and a positive predictive value of 82.6% in these patients [18]. The association between the presence of the wedge-sickle sign and clinical symptoms of GSH is highly significant (*p* < 0.0001) [18]. However, the diagnostic value of HRUS in patients without muscular atrophy is limited. In a study performed in 49 NTOS patients without a clear GSH, only 4 showed a wedge-sickle sign; the other 45 NTOS arms showed no abnormalities. Three of the four NTOS arms with a wedge-sickle sign were found to have a visible band, causing compression of the brachial plexus during thoracic outlet decompression (Figure 1) [46]. This study concluded that the incidence of objective findings in NTOS patients without GSH is too low to perform HRUS in every patient suspected of NTOS [46].

#### 6.3.5. Duplex for NTOS

Duplex ultrasound (DU) is suggested as a useful imaging technique because it can measure subclavian artery (SCA) compression in the thoracic outlet. Since the SCA and brachial plexus are closely related in the thoracic outlet (scalene triangle), SCA compression on DU is described by some as proof of brachial plexus compression and therefore helpful for diagnosing NTOS. Based on the same assumption, photo plethysmography (PPG) studies have been performed (and are still being performed) to diagnose NTOS and or ATOS. However, changes in flow velocities have been detected in both healthy patients and patients with NTOS [29]. The presence of ultrasound changes does not correlate with discoloration of the fingers or hand, treatment approach, or clinical outcome [29,47]. Therefore, we concluded that DU is not a reliable technique for diagnosing NTOS, and SCA compression should not be seen as confirmation of NTOS.

#### 6.3.6. Scalene Muscle Block (SMB)

Evidence exist that the outcome of an SMB can help in the diagnosis of NTOS and might predict surgical outcome [48]. The SMB in our center contains a local anesthetic injection into the anterior and medial scalene muscle to induce temporary muscle relaxation. If brachial plexus compression is related to muscular compression, an SMB can result in the temporary relief of symptoms. An increase in the successful postoperative relief of NTOS symptoms in patients with a positive SMB was reported by Sanders et al. [5]. This might be explained by the fact that these patients suffered from muscular compression of the brachial plexus. Considering both the diagnostic value as well as the prognostic value of an SMB, we performed such a block in all suspected NTOS patients.

In an attempt to improve the accuracy of the SMB measurement, we used sEAST both before and after the muscle block. The first measurement was performed to determine the patient’s baseline performance. Thereafter, an ultrasound-guided injection of 2 mL of bupivacaine in both the m. scalenus anterior and -medial on the affected side was performed. After 45–60 min, we performed a second sEAST measurement on the patient. The sEAST data of both measurements were compared before and after the bupivacaine injection and were considered positive if the duration of the sEAST increased >50 percent. Additionally, the patients were followed-up for three days after the muscle block in order to assess any subjective improvement using a Likert questionnaire that was digitally incorporated into their medical charts. Although not described previously, the Likert scale was introduced because we found that the patients delayed the reporting of symptom recurrence after a scalene muscle block which persisted for several days despite the short half-life value of bupivacaine.

## 7. Multidisciplinary Meeting

After completion of the diagnostic TOS care pathway, each patient was discussed in a multidisciplinary meeting. All the involved specialists in the care pathway were present and discussed each case individually in order to formulate a treatment plan. Only patients with insufficient progress after a specialized program of physical therapy were considered for TOD surgery.

### Thoracic Outlet Decompression Surgery (TOD)

In our center, thoracic outlet decompression (TOD) surgery was performed using a transaxillary approach (TA-TOD), supraclavicular approach (SC-TOD), and paraclavicular approach (PC-TOD). In all approaches, we strived for a complete first rib resection from sternocostal cartilage into the costovertebral joint, surpassing and dividing the ligament of the tubercle and anterior costotransverse ligament. It is of great importance to remove the rib from cartilage to cartilage since redo surgery is mostly conducted on incomplete rib resections that have regrown as part of the first ribs [49,50].

We chose a TA-TOD approach in most NTOS cases. In case of evident upper brachial plexus involvement, we chose an SC-TOD approach. The ideal approach for primary NTOS surgery is still a matter of debate [51,52,53].

Whether HRUS can help us to make a better choice regarding which approach is best suited for a primary operation is under investigation. During TA-TOD surgery, we also perform a distal scalenectomy, which involves the removal of the scalene muscles that intertwine through the brachial plexus. We believe that the removal of these scalene muscles results in the relief of compression, therefore relieving NTOS complaints. The first rib or scalenus muscles are not always the primary source of compression. Multiple forms of possible brachial plexus compression can be seen during surgery, such as fibromuscular adhesions, which typically compress the truncus inferior. TOD surgery should be patient-specific, in which the TOS surgeon removes all the compressive elements accordingly, including both bony and fibromuscular anomalies.

The SC-TOD approach is used for almost all redo cases. We find a dorsal first rib remnant with surrounding fibrotic scar tissue encompassing the T1 nerve and/or the truncus inferior of the brachial plexus in the majority of redo cases. This is in line with the literature, which states the importance of a complete first rib resection [49,54]. The PC-TOD approach is reserved for extensive redo surgery and vascular reconstructions, which are beyond the scope of this article.

## 8. Postoperative Rehabilitation Program

The postoperative rehabilitation program includes a strict physiotherapy program that is given to the patient on paper. Furthermore, instructions are given to the patient on the surgical ward on their day of discharge (which is in almost all cases the day after surgery). Patients are advised to wear a sling for 3 weeks after surgery. Physical therapy is provided to help patients achieve a full range of active motion (ROM) in 4–6 weeks, though not all patients achieve this in the first 6 weeks. Strenuous physical exercise should be avoided in the first 3 months after surgery. After 3 months, the physical therapist will gradually guide the patient back to his/her full strength. If necessary, physiotherapists from all over the Netherlands (as well as physiotherapists treating patients from abroad) can consult one of our physiotherapists, since it appears that knowledge of the specifics of the rehabilitation program of the NTOS population is scarce, which could result in unnecessary complications/recurrent symptoms after surgery.

## 9. Conclusions

NTOS is a challenging syndrome to diagnose. The NTOS diagnosis remains largely based on pattern recognition, physical examination, and the exclusion of differential diagnoses. Using imaging techniques in this care pathway is possible based on the existing literature, but their use remains partially based on expert opinion and is specific to each center. A single diagnostic tool for diagnosing NTOS does not exist, and one is also not expected to be developed in the upcoming years. More systematic and thorough research needs to be executed in order to find a single diagnostic tool of sufficient predictive value to diagnose NTOS.

## Figures and Tables

**Table 1:** Differential diagnoses for diagnosing neurogenic thoracic outlet syndrome (in alphabetical order).

Neurological	Musculoskeletal System	Vascular/Lymphogenic	Other
Brachial plexus lesion: - Traumatic - Malignancy - Latrogen	AC joint arthritis	Arm vein thrombosis caused by malignancy or catheter	Complex regional pain syndrome
Carpal tunnel syndrome	Biceps tendinopathy	ATOS	Pancoast tumor
Cervical radiculopathy	Cervical arthritis	Lymphoedema	Psychogenic pain
Multiple sclerosis	Costochondritis	Raynaud’s disease	Rheumatoid arthritis
Neuralgic amyotrophy (Parsonage–Turner)	Fibromyalgia	VTOS	Scleroderma
Ulnar neuropathy	Rotator cuff tendinopathy		
	Scapular dyskinesia		
	Subacromial syndrome		

## Data Availability

Data sharing is not applicable.
